# Subcutaneous advanced glycation end products, cardiovascular risk factors and vascular health during childhood development in a Swiss population

**DOI:** 10.3389/fphys.2024.1371618

**Published:** 2024-07-19

**Authors:** Christoph Hauser, Giulia Lona, Sabrina Köchli, Lukas Streese, Denis Infanger, Oliver Faude, Henner Hanssen

**Affiliations:** Department of Sport, Exercise and Health, Medical Faculty, University of Basel, Basel, Switzerland

**Keywords:** advanced glycation end products, retinal vessel diameters, pulse wave velocity, childhood cardiovascular risk, primary prevention

## Abstract

**Background:**

Skin-derived advanced glycation end products (sAGEs) have been associated with cardiovascular (CV) risk and mortality in adults. We hypothesize that cardiorespiratory fitness (CRF), body mass index (BMI) and vascular health are associated with development of sAGEs during childhood.

**Methods:**

In our prospective cohort study, 1171 children aged 6–8 years were screened for sAGEs, BMI, retinal arteriolar diameters (CRAE) and pulse wave velocity (PWV), using standardized procedures. To determine CRF a 20 m shuttle run was performed. After four 4 years, all parameters were assessed in 675 children using the same protocols.

**Results:**

Higher initial CRF levels were significantly associated with lower sAGEs (β [95 CI] −0.02 [−0.03 to −0.002] au, *p* = 0.022) levels at follow-up, although they showed a greater change from baseline to follow-up (β [95 CI] 0.02 [0.002 to 0.03] au, *p* = 0.027). Moreover, individuals with higher sAGEs at baseline showed narrower CRAE (β [95% CI] −5.42 [−8.76 to −2.08] μm, *p* = 0.001) at follow-up and showed a greater change in CRAE (β [95% CI] −3.99 [−7.03 to −0.96] μm, *p* = 0.010) from baseline to follow-up.

**Conclusion:**

Exercise and higher CRF may help mitigate the formation of AGEs during childhood, thereby reducing the risk for development of CV disease associated with AGEs-induced damage. Preventive strategies may need to target CRF early in life to achieve improvement of CV risk factors and may counteract the development of CV disease later in life.

## Introduction

Advanced glycation end products (AGEs) refer to a diverse assemblage of molecules resulting from the non-enzymatic glycation and oxidation of proteins, lipids, and nucleic acids (Thorpe and Baynes, 2003). These compounds induce changes in tissue functionality and mechanical characteristics through the formation of intermolecular connections among intracellular and extracellular matrix proteins (Kent et al., 1985; Brownlee et al., 1988; Sell et al., 1992; Bidasee et al., 2003; Bidasee et al., 2004). Through their interaction with the cell surface receptor, known as the receptor for AGEs (RAGE), they possess the capacity to regulate a multitude of cellular mechanisms (Wautier and Guillausseau, 1998; Min et al., 1999; Wautier et al., 2001). The accumulation of AGEs is associated with the development or exacerbation of many degenerative processes or diseases (Ahmad et al., 2018) such as diabetes (Singh et al., 2001) and cardiovascular (CV) diseases (Rasool et al., 2019). The mechanisms are multifactorial and include the pathology of oxidative stress and accelerated aging processes (Rungratanawanich et al., 2021; Chen C-y et al., 2022). The formation of AGEs is influenced by endogenous and exogenous factors. Briefly, endogenous factors include chronic inflammation, hyperglycemia, oxidative stress and the overproduction of reactive oxygen species (ROS), while exogenous factors include, for axample, the consumption of highly processed foods (Chen C-y et al., 2022).

Extensive research has been conducted on the skin, a tissue known for formation and build-up of AGEs. The existence of AGEs within the skin has been proposed as an indicator and predictor for the advancement of chronic cardiometabolic diseases (Meerwaldt et al., 2004; Monnier et al., 2005). Furthermore, concentrations of AGEs detected in the blood serum exhibit a good correlation with the accumulation of AGEs in the skin (Meerwaldt et al., 2004). Numerous studies have provided evidence supporting the association between skin autofluorescence (SAF)-derived AGEs (sAGEs) and the occurrence of CV morbidity and mortality in individuals with diabetes and end-stage renal failure (Meerwaldt et al., 2005; Lutgers et al., 2006; Gerrits et al., 2008). Additionally, there have been documented associations between elevated sAGEs levels and carotid intima thickness (Lutgers et al., 2010) as well as peripheral artery disease (De Vos et al., 2013; de Vos et al., 2014). In a recent meta-analysis conducted on adults with a high risk of CV events, it has been demonstrated that the presence of AGEs in the skin holds predictive value for both CV- and all-cause mortality (Cavero-Redondo et al., 2018). Chen et al. recently conducted a meta-analysis on individuals aged 18 years and older, demonstrating a significant correlation between elevated levels of AGEs, as quantified by SAF, and an increased aggregated risk of major adverse cardiovascular events (Chen Q. et al., 2022). A sedentary lifestyle and an imbalanced diet have been linked to the accumulation of AGEs in adults (Kim et al., 2017). Conversely, lifelong engagement in regular physical activity (PA) has been shown to counteract the age-related build-up of AGEs (Couppé et al., 2014). In adults, data on the association of body mass index (BMI) with sAGEs are conflicting, with some results showing an independent positive correlation and others showing a correlation only in the presence of the metabolic syndrome or no correlation at all (van Waateringe et al., 2017; van Waateringe et al., 2016; den Engelsen et al., 2012). There is a paucity of studies examining AGEs in children and adolescents. Childhood and adolescent obesity have previously been linked to reduced plasma levels of AGEs (Šebeková et al., 2009; Garay-Sevilla et al., 2018). However, elevated serum levels of AGEs have been reported in children at initial diagnosis of diabetes mellitus, implying their potential role in the progression of long-term complications (Jaisson et al., 2016). Furthermore, it has been reported that children exposed to diabetes for a duration of 5 years show an augmented accumulation of AGEs derived from SAF, reaching levels similar to those observed in healthy adults and corresponding to a 25-years advanced biological aging (Shah et al., 2013). Our baseline results of the EXAMIN YOUTH study demonstrated an association of sAGEs with cardiorespiratory fitness (CRF) but not with BMI (Köchli et al., 2020). The current large-scale longitudinal study with a follow-up of 4 years, aimed to assess the association of CRF and BMI with development of sAGEs during childhood development. We further aimed to investigate whether sAGEs-levels were associated with microvascular health and large artery stiffness. We hypothesized that higher CRF as well as healthy BMI have a favorable influence on the development of sAGEs over the investigation period and that higher levels of sAGEs are unfavorable associated with vascular health. The aim of our population-based clinical study in the school setting focused on the non-invasive assessment of sAGEs, but the assessment of molecular mechanisms and assessment of protein expression from serum markers was beyond the scope of the study.

## Materials and methods

### Study design and participants

**FIGURE 1:**
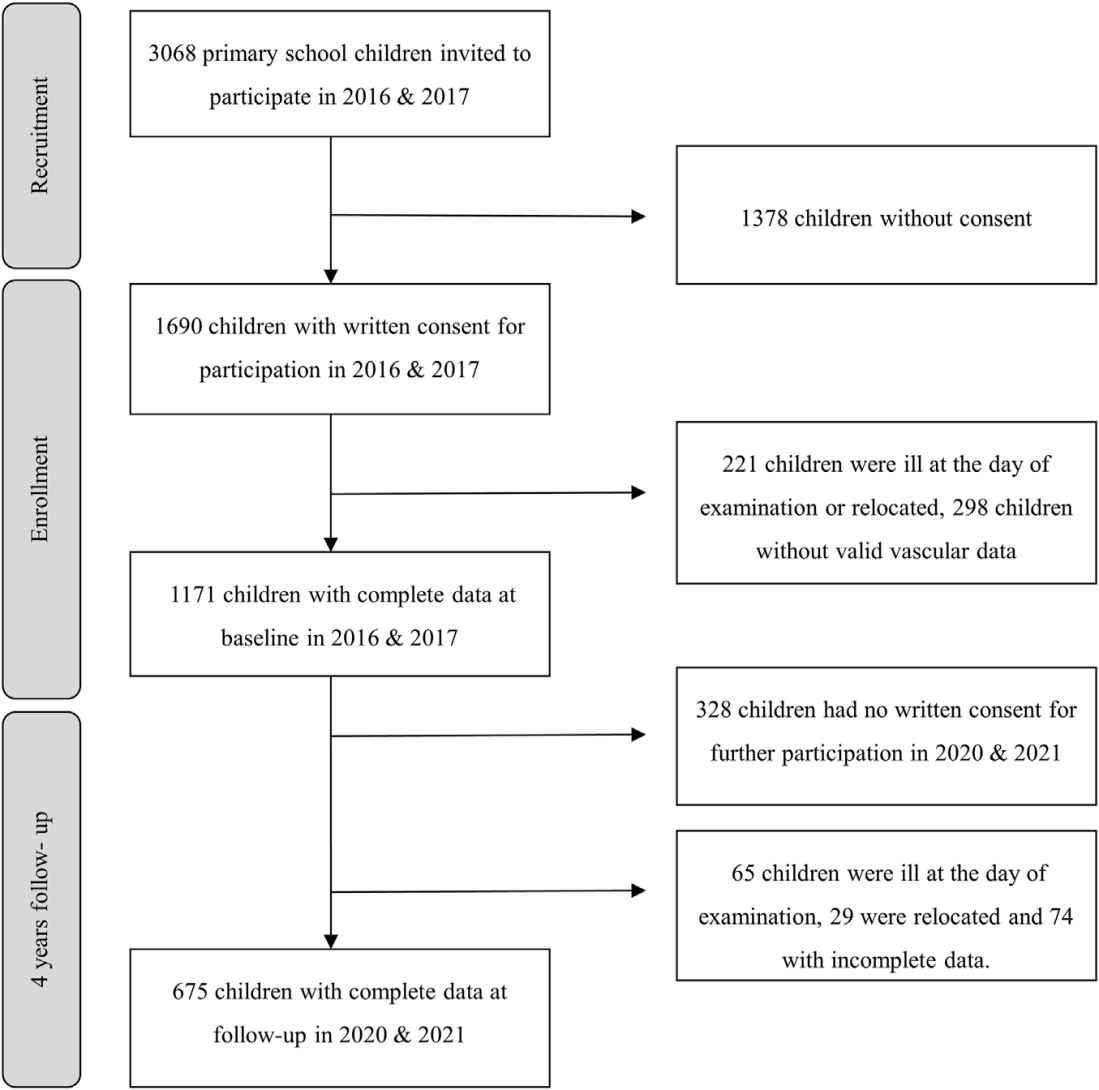
Flow-chart.

In 2016/2017, comprehensive baseline data were collected from all elementary schools in Basel, Switzerland. The study enrolled children aged between six and 8 years, whose parents had provided consent for medical screening. The medical assessments were conducted during regular class hours in the morning, with the children in a fasted state. The focus of the medical screenings was on blood pressure and vascular health. Additionally, anthropometric measurements and assessments of CRF were mandatory for all children and performed on a separate day during regular physical education lessons. Four years later, follow-up examinations were carried out under identical conditions (Figure 1). The results of the baseline analyses have been previously published (Köchli et al., 2020). The study obtained approval from the Ethics Committee of Northwestern and Central Switzerland (EKNZ, No. 258/12) and was registered on ClinicalTrials.gov (http://www.clinicaltrials.gov/: NCT02853747). The study adhered to the guidelines for good clinical practice and followed the Strengthening the Reporting of Observational Studies in Epidemiology statement (Association, 2001).

### Measurements

The same devices and standardized procedures were applied at baseline in 2016/17 and at follow-up in 2020/2021 to ensure standardization of individual changes over time. All measurements were performed by trained scientific staff.

### Cardiorespiratory fitness

The 20-m shuttle run (SR) is an established and dependable assessment method used to estimate maximal oxygen uptake in children with a high degree of validity (Van Mechelen et al., 1986; Léger et al., 1988). The procedure involved participants being given explicit instructions to traverse a distance of 20 m by running back and forth between two parallel lines, while maintaining synchronization with audio-based pacing signals. The test commenced at an initial running speed of 8 km/h and subsequently increased by 0.5 km/h every minute. The test terminated either when the participants reached a state of exhaustion or when they failed to reach the line twice consecutively within a range of 2 m. The score was determined by the number of lengths completed.

### Anthropometrics

Participants’ height was measured in an upright standing position, without wearing shoes, using a stadiometer (Seca, Basel, Switzerland). Weight measurements were obtained using a bioelectric impedance analyzer (InBody 170, Biospace device, InBody Co in Seoul, Korea), while participants were dressed in light sportswear and were barefoot. BMI was calculated by dividing the weight in kilograms by the square of the height in meters. To classify BMI values, age- and sex-specific reference values provided by Cole et al., 2000 were utilized. Participants with a BMI below the 85th percentile were classified as having normal weight, those falling between the 85th and 95th percentiles were categorized as overweight, and participants with BMI values above the 95th percentile were considered obese.

### Advanced glycation end products

Subcutaneous SAF as a method to assess AGEs was used. SAF measurements were carried out using the validated AGE Reader^®^ device (DiagnOptics Technologies BV, Groningen, Netherlands) (Meerwaldt et al., 2004; Meerwaldt et al., 2008). The AGE Reader^®^ device incorporates an integrated spectrometer that analyzes the reflected excitation light. By calculating the ratio between the emission light and reflected light, multiplied by 100, SAF values were obtained and expressed in arbitrary units (au). Emission light measurements were taken within the range of 420–600 nm, while the reflected excitation light fell within the range of 300–420 nm. SAF analysis provides a noninvasive and validated approach to evaluate AGEs in connective tissue, showing a strong correlation with AGEs accumulation in the blood serum (Meerwaldt et al., 2004). For further analysis, the mean value of three SAF measurements taken at different locations on the right ventral side of the forearm was utilized.

### Retinal vessel diameters and large artery pulse wave velocity

Retinal vessel analysis was performed using a fundus camera (Topcon TRC NW) and specialized analysis software (Visualis 3.0, iMEDOS Health GmbH, Jena, Germany). Trained scientific staff acquired two valid images of both eyes, with the optic nerve head positioned at the center, using a 45° angle. Subsequently, the diameters of retinal arterioles and venules were evaluated semi-automatically within a range of 0.5 to 1-disc diameter from the optic nerve head edge. This evaluation was conducted by two experienced examiners using Vesselmap 2 software (Visualis, iMEDOS Health GmbH), following established methods described previously (Streese et al., 2021). To determine average central retinal arteriolar diameter (CRAE) and central retinal venular diameter (CRVE), the Parr-Hubbard formula was applied (Hubbard et al., 1999). Additionally, the arteriolar-to-venular diameter ratio (AVR) was calculated using CRAE and CRVE (Hubbard et al., 1999). For consistency, the same vessels and vessel segments were marked in follow-up images to ensure optimal standardization, with the initial values from the baseline assessment serving as the reference for retinal analysis.

Central pulse wave velocity (PWV) was determined using the oscillometric Mobil-O-Graph monitor (I.E.M. GmbH, Germany) which has shown good agreement with the conventional tonometric method and has been validated for use in children (Wassertheurer et al., 2010; Mynard et al., 2020). The appropriate cuff size was selected based on the upper arm circumference and applied to the left arm of participants in a seated position. Following a rest period of 5 min, the device was calibrated using systolic blood pressure. At least two measurements were taken, with a 2-min interval between each measurement. Each measurement was carefully examined for quality, identifying any erroneous values, and repeated if necessary. Subsequently, the mean and standard deviation (SD) were calculated from at least two measurements with good quality.

### Statistical analysis

Population characteristics were described by calculating means and standard deviations (SD) for both baseline and follow-up data. A *t*-test for paired samples was conducted to compare the two sets of data. In order to assess potential selection bias, a *t*-test for independent samples was performed between the follow-up group and the lost-to-follow-up group. Partial correlation adjusted for age and systolic blood pressure (BP) was run to quantify the association between sAGEs with retinal vessel diameters and large artery pulse wave velocity. Pearson’s correlation was run to quantify the association between CRF and BMI. To investigate the association between CRF and BMI with sAGEs, changes in sAGE’s as well as retinal vessel diameters, and PWV, a linear mixed regression model was applied. Schools and classes nested within schools were included as random effects (West et al., 2006; Twisk et al., 2013). Directed acyclic graphs (DAGs) were employed to identify confounders necessary to minimize bias in the estimates (Tennant et al., 2021). Based on the DAG, models were adjusted for age, sex, socioeconomic status (SES), CRF, BMI and baseline vascular parameters. Multiple imputation using chained equation (MICE) was utilized to account for missing data of height, weight, BMI, and SES. Fifty datasets were imputed using predictive mean matching (White et al., 2011). The assumptions for regression models were assessed graphically using residual plots (Brown and Prescott, 2015). Marginal predicted means were used for graphic representation. The regression analyses were presented with β coefficients and corresponding 95% confidence intervals (CI). All tests were two-sided, and the significance level was set at 0.05. Stata 15 (StataCorp, College Station, TX, United States) was used for all calculation.

## Results

### Population characteristics

At baseline, a total of 1,171 children were assessed, and among them, 675 children had complete data at follow-up (Figure 1). The characteristics of the follow-up group were not significantly different from the lost-to-follow-up group (42%) in terms of height, weight, AVR, CRAE, CRVE, PWV, BP, BMI. However, the follow-up group had significantly higher CRF (31.1 vs. 28.9 lengths; *p* = 0.004) and lower sAGEs (0.98 vs. 1.04; *p* < 0.001) levels at baseline. Table 1 provides the absolute values and SD for the population characteristics at baseline, follow-up, and the mean differences over time. The prevalence of overweight and obesity at baseline was 9.4% and 2.8%, respectively. Over a period of 4 years, children experienced a significant increase in sAGEs (∆0.47 ± 0.01 au), BMI (∆2.5 ± 2.1 kg/m^2^), systolic BP (∆5 ± 9.4 mmHg), and PWV (∆0.3 ± 0.3 m/s). Furthermore, compared to baseline, children at follow-up exhibited significantly narrower CRAE (∆-7.2 ± 8.0 μm), CRVE (∆-1.4 ± 8.8 μm), and a lower AVR (∆-0.02 ± 0.04) (Table 1). It is worth noting that girls consistently exhibited wider CRAE and CRVE in comparison to boys at both time points. Furthermore, boys had statistically significant higher CRF and AGEs levels than girls at both time points, as indicated by the data presented in Table 1. Furthermore, we found an inverse correlation between CRF and BMI at baseline (r_s_ = −0.33, *p* < 0.001) and follow-up (r_s_ = −0.45, *p* < 0.001).

**TABLE 1:** Population characteristics at baseline and follow-up.

Parameter	2016/2017			2020/2021			Difference	
	N	Mean	SD	N	Mean	SD	Mean	SD
Sex (female, %)	391 (51.86%)							
Age, y	747	7.2	0.36	747	11.4	0.4	4.2	0.2
BMI, kg/m^2^	736	15.7	2.0	544	18.2	3.4	2.5	2.1
Underweight^a^	124 (17.80%)			103 (18.93%)				
Normal^a^	514 (70.80%)			362 (66.54%)				
Overweight^a^	68 (9.37%)			62 (11.40%)				
Obese^a^	20 (2.75%)			17 (3.12%)				
Height, cm	727	124.5	5.4	538	149.4	7.2	24.9	4.3
Weight, kg	726	24.5	4.5	544	40.9	9.8	16.4	6.6
CRF, lengths	720	31.1	12.3	533	49.1	19.3	17.3	16.1
Male	344	34.4^*^	13.1	254	55.1^*^	20.3		
Female	376	28.2^*^	10.8	279	43.5^*^	16.5		
SBP	749	104	7.8	749	109	9.8	5	9.4
DBP	749	64	6.8	749	65	7.8	1	8.2
sAGEs, au	668	0.98	0.21	675	1.45	0.69	0.47	0.01
Male, au	315	1.02^*^	0.02	320	1.48^*^	0.01		
Female, au	353	0.97^*^	0.01	355	1.42^*^	0.01		
CRAE, µm	694	202.6	12.8	724	195.4	12.3	−7.2	8.0
Male, µm	339	200.1^*^	12.4	346	192.7^*^	11.8		
Female, µm	335	205.0^*^	12.7	378	197.8^*^	12.3		
CRVE, µm	694	229.6	13.9	724	228.2	13.9	−1.4	8.8
Male, µm	339	227.9^*^	14.1	346	226.1^*^	13.5		
Female, µm	355	231.3^*^	13.6	378	230.1^*^	14.1		
AVR	694	0.88	0.05	724	0.86	0.05	−0.02	0.04
Male	339	0.87	0.05	346	0.85^*^	0.05		
Female	355	0.88	0.05	378	0.86^*^	0.05		
PWV, m/s	664	4.3	0.3	729	4.6	0.3	0.3	0.3
Male, m/s	322	4.3	0.3	346	4.6	0.3		
Female, m/s	342	4.3	0.3	383	4.6	0.3		

sAGEs, skin autofluorescence-derived advanced glycation end products; au, arbitrary units; AVR, arteriolar-to-venular diameter ration; BMI, body mass index; CRAE, central retinal arteriolar equivalent; CRF., cardiorespiratory fitness; CRVE, central retinal venular equivalent; DBP, diastolic blood pressure; PWV, pulse wave velocity; SBP, systolic blood pressure.

^a^ According age- and sex-specific reference values provided by Cole et al.

^*^Indicates a significant difference between sex.

### Cardiorespiratory fitness, body mass index and development of AGEs

**FIGURE 2:**
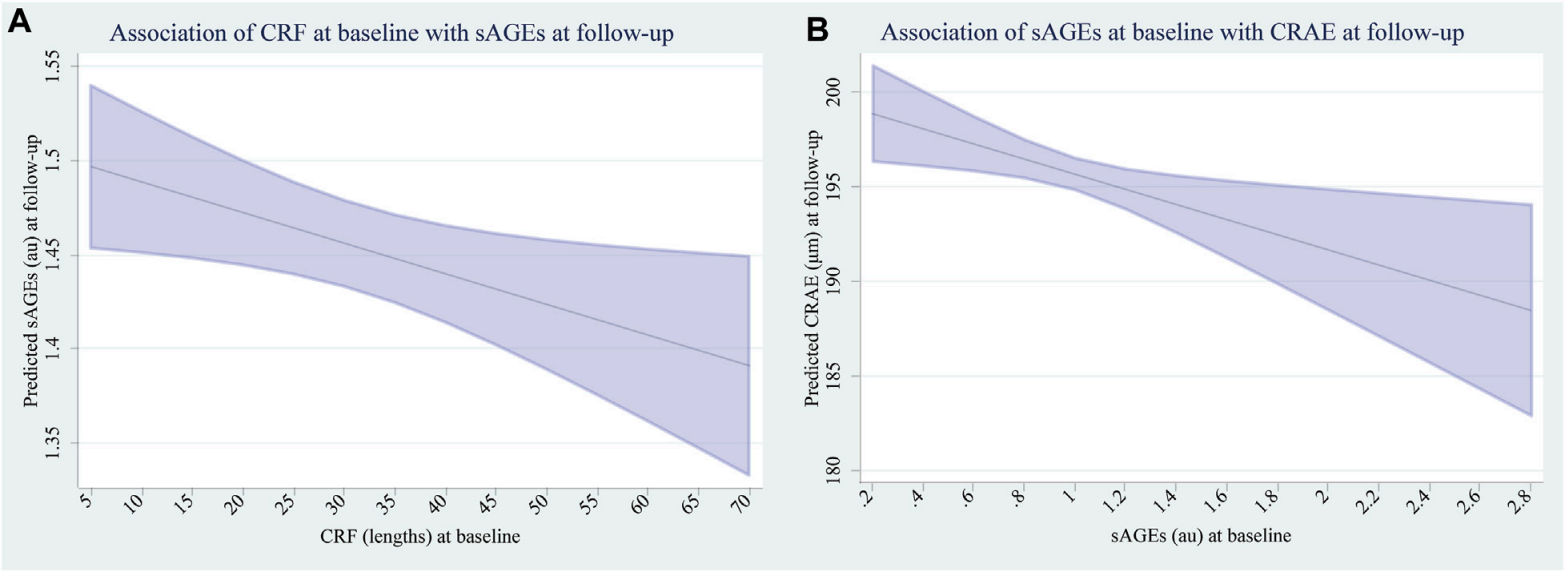
**(A)** Marginal predicted means of sAGEs at follow-up based on CRF at baseline. **(B)** Marginal predicted means of CRAE at follow-up based on sAGEs at baseline.

The associations between CRF and BMI at baseline with subcutaneous AGEs after 4 years are presented in Table 2. Children with higher CRF at baseline developed significantly lower sAGEs level (β [95 CI] −0.02 [−0.03 to −0.002] au decrease per additional 10 lengths in SR, *p* = 0.022). The corresponding plot with marginal predicted means of AGEs at follow-up based on CRF at baseline is shown in Figure 2A. We found no evidence for an association between baseline BMI and sAGEs level at follow up (β [95 CI] 0.007 [−0.002 to 0.002] au per 1 kg/m^2^ increase, *p* = 0.139). The association between CRF and BMI at baseline with changes in subcutaneous AGEs over the 4-year period are presented in Table 2. Children with higher CRF at baseline demonstrated a significantly greater change in sAGEs (β [95 CI] 0.02 [0.002 to 0.03] au increase per additional 10 lengths in SR, *p* = 0.027) over the period of 4 years. Initial BMI was not associated with changes in subcutaneous AGEs level (β [95 CI] 0.007 [-0.003 to 0.02] au increase per 1 kg/m^2^ increase, *p* = 0.179) concentration over the study period. A subgroup analysis, in which the children were divided into two groups based on their baseline CRF (<25th percentile & >25th percentile), showed a significant difference between the groups in terms of mean sAGEs concentration (−0.09 (−0.11 to −0.08), *p* < 0.001). We found no evidence for an accelerated change in sAGEs (β [95 CI] 0.4 [0.02 to 0.8] au increase per additional 10 lengths in SR, *p* = 0.062) over the 4-year period in children with initially poor fitness (data not shown).

**TABLE 2:** Association of Cardiorespiratory Fitness and Body Mass Index with sAGEs.

Parameter	sAGEs at follow-up (au decrease per additional 10 lengths in SR)	
	β (95% CI)	*p*-Value
CRF at baseline (lengths in SR)^a^	−0.02 (−0.03 to −0.002)	0.022

Parameter	sAGEs at follow-up (au increase per unit)	
	β (95% CI)	*p*-Value
BMI at baseline (kg/m^2^)^b^	0.007 (−0.002–0.02)	0.139

Parameter	∆ sAGEs (au decrease per additional 10 lengths in SR)	
	β (95% CI)	*p*-Value
CRF at baseline (lengths in SR)^a^	0.02 (0.002–0.03)	0.027

Parameter	∆ sAGEs at (au increase per unit)	
	β (95% CI)	*p*-Value
BMI at baseline (kg/m^2^)^b^	0.007 (−0.003–0.02)	0.179

sAGEs, skin autofluorescence-derived advanced glycation end products; BMI, body mass index; CRF, cardiorespiratory fitness; SR, shuttle run.

^a^ Adjusted for sex, SES, at baseline and age at follow-up.

^b^ Adjusted for sex, SES, CRF, at baseline and age at follow-up.

### AGEs and development of vascular health

The association between subcutaneous AGEs at baseline with development of micro- and macrovascular health at follow-up are presented in Table 3. Children with higher initial sAGEs levels developed significantly narrower CRAE over the follow-up period (β [95% CI] −3.99 [−7.03 to −0.96] μm decrease per 1 au, *p* = 0.010). The corresponding plot with marginal predicted means of CRAE at follow-up based on sAGEs at baseline, is shown in Figure 2B. We found no evidence for the association between sAGEs at baseline and CRVE (β [95% CI] −1.49 [−4.87 to 1.88] μm decrease per 1 au, *p* = 0.386), AVR (β [95% CI] −0.01 [−0.03 to 0.001] units decrease per 1 au, *p* = 0.070) and PWV (β [95% CI] 0.02 [−0.07 to 0.12] m/s increase per 1 au, *p* = 0.658) at follow-up. The association between sAGEs at baseline and changes of micro- and macrovascular health are presented in Table 3. Children with initially higher sAGEs levels demonstrated a greater change in CRAE (β [95% CI] −5.42 [−8.76 to −2.08] μm decrease per 1 au, *p* = 0.001). We found no evidence for the association between sAGEs at baseline and changes in CRVE (β [95% CI] −2.98 [−6.63 to 0.66] μm decrease per 1 au, *p* = 0.109), AVR (β [95% CI] −0.01 [−0.27 to 0.02] units decrease per 1 au, *p* = 0.088) and PWV (β [95% CI] 0.07 [−0.07 to 0.2] m/s increase per 1 au, *p* = 0.010).

**TABLE 3:** Association of sAGEs at Baseline with Development of Vascular Health.

Parameter	CRAE at follow-up (µm change per unit increase)		CRVE at follow-up (µm change per unit increase)		AVR at follow-up (Units change per unit increase)		PWV at follow-up (m/s change per unit increase)	
	β (95% CI)	*p*-Value	β (95% CI)	*p*-Value	β (95% CI)	*p*-Value	β (95% CI)	*p*-Value
sAGEs at baseline (au)^a^	−3.99 (−7.03 to −0.96)	0.010	−1.49 (−4.87 to 1.88)	0.386	−0.01 (−0.03 to 0.001)	0.070	0.02 (−0.07–0.12)	0.658

Parameter	∆ CRAE (µm change per unit increase)		∆ CRVE (µm change per unit increase)		∆ AVR (Units change per unit increase)		∆ PWV (m/s change per unit increase)	
	β (95% CI)	*p*-Value	β (95% CI)	*p*-Value	β (95% CI)	*p*-Value	β (95% CI)	*p*-Value
sAGEs at baseline (au)^b^	−5.42 (−8.76 to −2.08)	0.001	−2.98 (−6.63 to 0.66)	0.109	−0.01 (−0.27 to 0.002)	0.088	0.07 (−0.07–0.2)	0.329

sAGEs, skin autofluorescence-derived advanced glycation end products; AVR, indicates arteriolar-to-venular diameter ration; BMI, body mass index; CRAE, central retinal arteriolar equivalent; CRVE, central retinal venular equivalent; PWV, pulse wave velocity.

^a^ Adjusted for sex, vascular parameter at baseline (CRAE, CRVE, AVR, or PWV) and SBP, age at follow-up.

^b^ Adjusted for sex at baseline and SBP, age at follow-up.

### Interrelation of sAGEs with vascular biomarkers

The partial correlations between subcutaneous AGEs and retinal vessel diameters as well as large pulse wave velocity adjusted for age and systolic BP are presented in Table 4. At baseline in children aged six to eight, we found no correlation between sAGEs and micro- or macrovascular health. Four years later, we found a significant, albeit weak inverse correlation between sAGEs and AVR (r = −0.12, *p* = 0.002) and a weak positive correlation between sAGEs and CRVE (r = 0.11, *p* = 0.008) as well as PWV (r = 0.072, *p* = 0.041).

**TABLE 4:** Partial Correlation between AGEs and Vascular Health adjusted for age and systolic Blood Pressure.

Baseline								
	CRAE		CRVE		AVR		PWV	
	r	*p*-Value	r	*p*-Value	r	*p*-Value	r	*p*-Value
sAGEs	0.02	0.572	0.04	0.271	−0.02	0.693	−0.02	0.569

Follow-up								
	CRAE		CRVE		AVR		PWV	
	r	*p*-Value	r	*p*-Value	r	*p*-Value	r	*p*-Value
sAGEs	−0.0007	0.864	0.11	0.008	−0.12	0.002	0.07	0.041

sAGEs, skin autofluorescence-derived advanced glycation end products; AVR, indicates arteriolar-to-venular diameter ration; BMI, body mass index; CRAE, central retinal arteriolar equivalent; CRVE, central retinal venular equivalent; PWV, pulse wave velocity; r, partial correlation adjusted for age and systolic BP.

## Discussion

As main findings, higher initial CRF levels were associated with lower subcutaneous AGEs levels at follow-up during childhood development. Moreover, children with higher sAGEs levels at baseline showed unfavorable microvascular health at follow-up, indicated by narrower CRAE and a greater change in CRAE from baseline to follow-up. Additionally, after adjustment for systolic BP and age, there was no correlation between sAGEs and any vascular parameter at the study’s outset. However, during the follow-up, there was a weak positive correlation between sAGEs and CRVE as well as PWV, and a weak inverse correlation between sAGEs and AVR.

Our results imply that CRF affects levels of sAGEs and can predict the progression of sAGEs levels in children over 4 years. We have previously observed similar results in our cross-sectional study, where children with higher CRF demonstrated lower levels of sAGEs compared to those with lower CRF (Köchli et al., 2020). These findings are consistent with a review in adults, which emphasized the beneficial effects of regular PA on AGEs levels (Alyafei et al., 2020). Therefore, it is plausible that regular PA and improved CRF may have a protective effect against the endogenous accumulation of AGEs. Physical activity and increased CRF have been shown to increase insulin sensitivity, reduce inflammation and improve overall metabolic function and health (Schuler et al., 2013). These mechanisms may contribute to reduced production or increased clearance of AGEs, resulting in lower AGEs levels in individuals with higher CRF. Furthermore, one could postulate that children who are fitter and more active in their daily lives may come from households that are more likely to focus on a healthy lifestyle and healthy nutrition and, consequently, consume fewer highly processed foods. However, we did not find a significant association between baseline BMI and sAGE levels at follow-up. The existing data on the association between BMI and subcutaneous AGEs is inconsistent as mentioned previously (van Waateringe et al., 2017; van Waateringe et al., 2016; den Engelsen et al., 2012; Köchli et al., 2020). A recent meta-analysis involving nine studies conducted in adults revealed a negative correlation between obesity and the levels of soluble advanced glycation end product receptor (sRAGE). sRAGE serves as a natural antagonist, binding to AGEs and inhibiting their interaction with RAGE, thereby preventing the activation of the detrimental signaling cascade (Huan et al., 2022). However, a study focused specifically on adolescents with obesity, found significantly lower levels of sRAGE in the obese group compared to the normal-weight group (Rodríguez-Mortera et al., 2019). Conversely, Sebekova et al. did not find a statistically significant difference in sRAGE levels between normal weight and children with obesity (Šebeková et al., 2009). While BMI is commonly used as an indicator of obesity, it may not capture the specific metabolic and physiological factors that influence the accumulation of AGEs. Other factors such as body fat distribution, insulin resistance or dietary habits might play a more significant role in determining AGE levels, especially in populations with a short duration of risk exposure such as children.

To the best of our knowledge, this is the first study to examine the potential link between sAGEs and development of vascular health in children over 4 years. Notably, we have shown that higher level of subcutaneous sAGEs at baseline were significantly associated with narrower CRAE at follow-up. Furthermore, initial levels of sAGEs were associated with a greater change in CRAE over the investigation period. These results suggest that sAGEs can serve as a predictive indicator for the narrowing of CRAE over time. In adults with existing diseases, previous cross-sectional studies have demonstrated a significant association between higher levels of subcutaneous AGEs and narrower CRAE, lower AVR as well as higher PWV (Llauradó et al., 2014; van Eupen et al., 2016; Vaes et al., 2020). In older adults, narrower arteriolar and wider venular diameters have been associated with severity of hypertension (Ikram MK. et al., 2006), increased risk of stroke (Ikram M. et al., 2006; McGeechan et al., 2009), and increased CV mortality (Wang et al., 2007). Furthermore, increased large artery stiffness has been shown to be an independent predictor for the risk of stroke as well as CV morbidity and mortality in the general population and in patients with CV disease (Laurent et al., 2001; Mattace-Raso et al., 2006; Willum et al., 2006; Vlachopoulos et al., 2010). The relation between AGEs and retinal vessel diameters as well as large artery stiffness is complex and multifactorial. AGEs engage in multi-level interactions within tissues, resulting in deleterious consequences such as remodeling of collagen in the vascular wall, alterations in calcium homeostasis and over all increased oxidative stress and inflammation. These interactions encompass cross-linking events with extracellular proteins like collagen and elastin, consequently altering the mechanical properties of the tissue (Zieman and Kass, 2004). Intracellularly, AGEs induce modifications in physiological properties (Bidasee et al., 2003; Bidasee et al., 2004). Furthermore, AGEs exhibit binding affinity towards the cell surface receptor RAGE, thus instigating a wide array of intracellular signaling cascades (Neeper et al., 1992). The observed results may be reasonably accounted for by the interaction between AGEs and their receptor, RAGE. This interaction initiates an intracellular cascade, which subsequently results in a reduction of nitric oxide (NO) bioavailability (Zieman and Kass, 2004; Hegab et al., 2012). Consequently, impaired endothelial function ensues, leading to constriction of the retinal arterioles. Interestingly, our investigation did not reveal a correlation between the initial levels of AGEs and PWV values during the follow-up period. One plausible explanation for this finding is that structural adaptations necessitate a longer duration than functional adaptations to become apparent. As a result, it can be postulated that the microcirculation, specifically the retinal arteriolar diameters, exhibits greater susceptibility to the effects of AGEs compared to the macrocirculation. Furthermore, after adjustment for systolic BP and age, we found no correlation between baseline sAGEs levels and any vascular parameter in children aged six to 8 years. Four years later, we found a weak positive correlation between AGEs and CRVE as well as PWV, and a weak inverse correlation with AVR. These results suggest that the influence of AGEs on vascular health in children may become more apparent over time. Nevertheless, these findings contribute to our understanding of potential impact of AGEs on the vascular system. Further research is warranted to elucidate the clinical significance of these associations.

Our study has some limitations. First and foremost, the effect sizes in our cohort of otherwise healthy young children appear small and their clinical relevance for development of CV risk and disease in adulthood need to be investigated in future long-term studies. Furthermore, the assessment of CRF in our study was conducted using the 20 m SR due to practical considerations and the school setting, which made spiroergometry impractical. Nonetheless, the 20 m SR test is a reliable and valid method for estimating maximal oxygen uptake in children (Van Mechelen et al., 1986; Léger et al., 1988). In our study, the measurement of AGEs concentration was performed using SAF rather than blood samples, which could have potentially impacted our findings. Nevertheless, it is important to note that SAF measurements have been shown to exhibit a strong correlation with AGEs concentrations in the blood (Meerwaldt et al., 2004). While it is acknowledged that not all AGEs possess inherent fluorescent properties, there is a possibility of other tissue components that fluoresce within the same wavelength range, which could introduce confounding factors. However, the SAF method has undergone validation procedures involving the assessment of specific AGE levels in skin biopsies obtained from both healthy individuals and patients diagnosed with diabetes (Meerwaldt et al., 2004). Pulse wave velocity was measured using an oscillometric device, a method strongly dependent on BP and age, rather than a tonometric carotid-femoral measurement for reason of practicability when screening children in school settings. Indeed, the oscillometric approach has been reported to underestimate arterial stiffness in younger subjects and to overestimate it with increasing age (Del Giorno et al., 2021). Moreover, it is important to acknowledge that our study did not include data regarding pubertal status and eating habits, both of which have the potential to exert an additional influence on our results. It is important to note that our study was conducted within a predominantly Caucasian Swiss population, which limits the generalizability of our results to other ethnic groups. Additionally, the progression of our follow-up research was disrupted by the COVID-19 pandemic. The implementation of pandemic-related restrictions, such as temporary school closures and limitations in the built environment, may have had an impact on physical activity patterns and overall wellbeing, potentially influencing the outcomes of our study. However, the prevalence of SARS-CoV-2 infection among Swiss schoolchildren remained relatively low, even during periods of high incidence in the general population. Moreover, the prevalence of asymptomatic cases among schoolchildren was also reported to be low (Kriemler et al., 2021). These factors suggest that direct effects of SARS-CoV-2 infection on the collected data are unlikely.

## Conclusion

Overall, our findings underscore the critical role of maintaining high levels of CRF in mitigating the pathophysiological impacts of AGEs during childhood development. Engaging in regular physical activity, consistent exercise and maintaining a healthy weight and diet may help to counteract the endogenous and exogenous formation of AGEs, thereby reducing the risk for development of chronic disease associated with AGEs-induced damage. Our results indicate the potential of non-invasive sAGEs assessment for potential clinical implication to improve primary prevention during childhood. These measurements can help identify individuals at risk at an early stage and enable early treatment initiation. While our study contributes to the understanding of the potential impact of AGEs on vascular health in children, further research is needed to fully comprehend the clinical significance of these associations. Future studies should explore the long-term effects of CRF, weight management and dietary habits on AGE accumulation, vascular function, and the development of chronic diseases. By elucidating these relationships, we can enhance preventive strategies and interventions aimed at reducing AGE-induced damage and improving vascular health in both children and adults.

## Data Availability

The raw data supporting the conclusions of this article will be made available by the authors, without undue reservation.
